# Driving CAR T Stem Cell Targeting in Acute Myeloid Leukemia: The Roads to Success

**DOI:** 10.3390/cancers13112816

**Published:** 2021-06-05

**Authors:** Ilaria M. Michelozzi, Efstratios Kirtsios, Alice Giustacchini

**Affiliations:** Molecular and Cellular Immunology Section, UCL Great Ormond Street Institute of Child Health, Zayed Centre for Research into Rare Disease in Children, London WC1N 1DZ, UK; efstratios.kirtsios.18@ucl.ac.uk

**Keywords:** acute myeloid leukemia, leukemic stem cells, bone marrow niche, chimeric antigen receptor T-cells

## Abstract

**Simple Summary:**

Chimeric antigen receptor (CAR) T-cells are powerful therapeutic tools that have revolutionized the treatment of several hematological malignancies. However, their therapeutic application in acute myeloid leukemia (AML) remains challenging. In this review, the authors aimed to dissect how AML-leukemic stem cell and AML-bone marrow niche features can impact on the success of CAR T-cell therapy. The clinical implementation of some of the newly developed approaches discussed in this review may lead to the development of safe and effective CAR T-cell strategies for AML, accounting for the disease heterogeneity.

**Abstract:**

Current treatment outcome for acute myeloid leukemia (AML) patients is unsatisfactory and characterized by high rates of relapse and poor overall survival. Increasing evidence points to a crucial role of leukemic stem cells (LSC) and the bone marrow (BM) leukemic niche, in which they reside, in AML evolution and chemoresistance. Thus, future strategies aiming at improving AML therapeutic protocols are likely to be directed against LSC and their niche. Chimeric antigen receptor (CAR) T-cells have been extremely successful in the treatment of relapsed/refractory acute lymphoblastic leukemia and B-cell non-Hodgkin lymphoma and comparable results in AML are highly desirable. At present, we are at the dawn of CAR T-cell application in AML, with several preclinical studies and few early phase clinical trials. However, the lack of leukemia-specific targets and the genetic and phenotypic heterogeneity of the disease combined with the leukemia-induced remodeling of the BM microenvironment are limiting CAR T-cell exploitation in AML. Here, we reviewed AML-LSC and AML-BM niche features in the context of their therapeutic targeting using CAR T-cells. We summarized recent progress in CAR T-cell application to the treatment of AML, and we discussed the remaining therapeutic challenges and promising novel strategies to overcome them.

## 1. Introduction

Acute myeloid leukemia (AML) is a life-threatening blood cancer characterized by the uncontrolled/abnormal proliferation of myeloblasts that accumulate mainly in bone marrow (BM) and peripheral blood [1].

With a patient’s average age at diagnosis of 68 years [2], AML mostly affects adults.

Despite the recent progress made in AML diagnosis, risk stratification, and prognosis, treatments have not considerably changed over the last two decades. In particular, AML therapeutic pillars consist of an induction therapy, based on cytarabine and anthracycline, followed by a consolidation regimen, including chemotherapy and/or allogeneic stem cell transplantation, necessary to kill residual leukemic clones to prevent relapse [1,3]. Patients’ survival is extremely poor, especially for the elderly (≥65 years), only 30% of whom survive over 1 year post-diagnosis [1], with relapse rates ranging from 9% to 78% [4].

To date, the biological and clinical complexity of the disease, largely attributable to its molecular and phenotypic heterogeneity, hinders the development of a successful treatment for AML [5].

AML cell populations are organized in a hierarchical structure dominated by a rare and heterogeneous subset of cells [6,7,8], the so-called leukemic stem cells (LSC). LSC are capable of initiating and maintaining the disease [6] and have been shown to fuel disease relapse due to their intrinsic and extrinsic (BM microenvironment-mediated) chemoresistance properties [9,10,11].

Due to the central role of LSC in AML pathogenesis and progression, their therapeutic targeting and elimination are imperative in order to improve patient’s outcome [5].

Recently, the technological progress in high-throughput single-cell approaches offered an unprecedented resolution on tumor heterogeneity. Indeed, single-cell analyses provide a powerful tool not only to dissect clonal evolution and hierarchical structure of hematological disorders [12,13,14,15,16,17], but also to unravel the unique features of malignant stem cells and their BM niche dependencies [14,15,16,18,19]. Results from single-cell analyses have the potential to gain new insights into the pathogenesis of several hematological diseases and identify new candidate molecules for targeted therapies [12,14,15,16,18,19].

Novel therapies are currently under preclinical and clinical investigation in AML, as extensively described in [20,21,22], and they mainly aim at targeting LSC, the molecular processes altered in LSC and AML blasts, and their interplay with the BM microenvironment.

While recently developed small-molecule inhibitors have a role in subsets of AML (e.g., *IDH1/2* and *FLT3* mutated) [20], because of the genetic heterogeneity of this disease they are unlikely to be broadly applicable.

One strategy that is presently being explored with limited success [21] but with a potential broader applicability involves the application of chimeric antigen receptor (CAR) T-cells.

CAR T-cells, which uniquely combine the specificity of a monoclonal antibody with the efficacy of a cytotoxic T-cell, have revolutionized the treatment of several hematological malignancies, especially relapsed/refractory acute lymphoblastic leukemia (ALL) and B-cell non-Hodgkin lymphoma [23].

Comparable applications in AML would be highly beneficial. However, the development of immunotherapeutic strategies harnessing the power of engineered T-cells against AML has been hindered by the disease heterogeneity and complicated by the chemo-resistant and immune-evasive properties of AML-LSC and by the AML-BM niche characteristics.

Herein, we reviewed the role of AML-LSC and the leukemia-associated BM remodeling in treatment escape. We summarized the recent developments in the application of CAR T-cells to the treatment of AML and we discussed the main challenges ahead, as well as potential novel strategies to progress toward successful CAR T-cell therapy for AML.

## 2. The Role of LSC and the BM Microenvironment in AML

**Figure 1:**
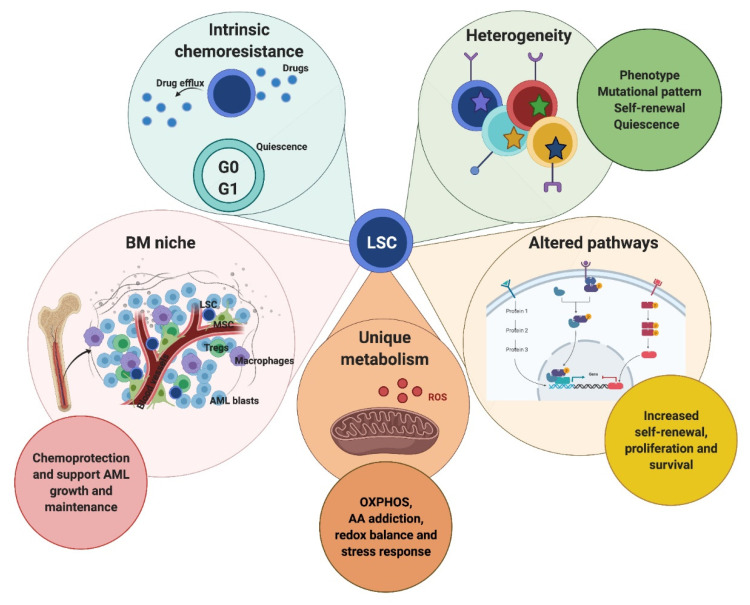
Summary of leukemic stem cell (LSC) distinctive features. LSC are characterized by phenotypic, mutational, self-renewal- and quiescence-related heterogeneity. This, in addition to intrinsic and extrinsic chemoresistance, could hamper acute myeloid leukemia (AML) treatment efficacy. The efflux pumps expressed on LSC surface and LSC quiescence determine their intrinsic drug resistance. Moreover, the AML-bone marrow (BM) niche represents a LSC cradle which supports AML cell expansion and survival providing chemoprotection. On the other hand, LSC exhibit unique metabolism and altered molecular pathways offering selective therapeutic targets. LSC, leukemic stem cells; BM, bone marrow; MSC, mesenchymal stromal cells; Tregs, regulatory T-cells; AML, acute myeloid leukemia; ROS, reactive oxygen species; OXPHOS, oxidative phosphorylation; AA, amino acids.

AML-LSC were first identified and functionally defined as the only cells capable of initiating and maintaining the disease in xenotransplantation settings, due to their unique self-renewal and proliferation properties [6,24]. Since then, AML-LSC biological and molecular features have been extensively studied to identify their therapeutic vulnerabilities and determine how they contribute to AML clinical complexity. LSC features are outlined in Figure 1.

In the next sections, we discuss some of the key factors challenging AML-LSC therapeutic targeting: (1) their similarity with normal hematopoietic stem cells (HSC), (2) their heterogeneity, and (3) the leukemia-induced remodeling of the BM niche.

### 2.1. LSC vs. HSC: Selective LSC-Targeted Eradication

Since the AML hierarchical structure was first described, parallelisms with normal hematopoiesis have been extensively investigated.

Like HSC, LSC mainly reside within the BM [25], are highly quiescent, and are able to self-renew as well as to give rise to more mature cell subsets [9].

Similarly to HSC, LSC display increased chemoresistance, due to their intrinsic properties (such as their quiescent state and the increased expression of components of multi-drug efflux pumps) or to the leukemia supportive BM microenvironment [9,25].

Furthermore, LSC and HSC share similar immunophenotypic patterns (e.g., CD13, CD33, CD71, CD99, CD117, CD133, CD200, CD244) and it is widely accepted that they both reside within the lineage negative (Lin−)CD34+CD38− compartment [21,26].

These shared features challenge the efficient discrimination between LSC (to be therapeutically targeted) and HSC (to be spared and rescued).

However, LSC display distinct antigenic, molecular, and metabolic traits that could render them selectively targetable.

Several surface antigens have been shown to be preferentially expressed in LSC against HSC and thus proposed as potential biomarkers and therapeutic targets (e.g., CD25, CD32, CD44, CD47, CD96, CD123, TIM3, CLL1) [27]. Some of these markers are currently being evaluated as CAR T-cell targets, as discussed in the next paragraph of this review.

Differential gene expression analyses have also revealed LSC-specific signatures, including genes involved in various pathways, such as adherens junction, actin cytoskeleton organization, apoptosis, and MAPK, JAK-STAT, and Wnt signaling pathways [28].

Recently, new analytical methods based on the simultaneous detection of mutations and whole transcriptome at the single-cell level have allowed unprecedented resolution in discriminating between normal and malignant single stem cells within the same tumor [14,15,16,18].

The application of one of these single-cell integrated approaches in the AML context has revealed novel LSC-specific candidate surface markers encoded by *CD36* and *CD74* genes. This approach also uncovered a “mixed” molecular signature specific to AML early progenitors and characterized by the simultaneous expression of genes associated with both stemness and myeloid priming as well as altered transcriptional programs with an increased expression of proliferation-, self-renewal-, stress response-, and redox signaling-related genes [16].

The increased survival, self-renewal, and expansion capacity of LSC, as compared to HSC, have been shown to be at least in part due to the constitutive activation of NF-KB, JAK-STAT, PI3K/AKT/mTOR pathways, to the deletion of tumor suppressor genes (e.g., *PTEN*), to the upregulation of anti-apoptotic genes, and to alterations in Wnt/β-catenin, Hedgehog, and Notch signalings and in microRNA levels (e.g., miR-9 and miR-126) reported in LSC [9,29].

Metabolically, although LSC and HSC are both marked by small amounts of reactive oxygen species (ROS) [30], LSC display distinctive features, including their higher dependence on oxidative phosphorylation and on regulators of mitochondrial functionality, redox balance, and response to stress (e.g., mitophagy-associated proteins including FIS1, the pro-survival protein BCL-2, a cancer-specific heat shock protein species teHsp90, unfolded protein response and, the above mentioned, NF-KB pathway) for their preservation [5,30]. Moreover, LSC preferential addiction to amino acid metabolism and enhanced sensitivity to variations in amino acid availability have also been reported [31,32].

Altogether the characterization of dysregulated pathways in LSC has improved since their discovery, the lack of exclusive targetable antigens still limits the development of safe and effective clinical protocols directed against LSC. The application of single-cell technologies to the identification of novel targets in LSC promises to revolutionize the development of targeted therapies.

### 2.2. Sources of Heterogeneity in AML-LSC

AML is a molecularly heterogeneous group of diseases with a complex mutational landscape. Recently, over 5000 driver mutations across 76 genes or genomic regions were identified, with 2 or more drivers present in 86% of reported cases [33]. AML heterogenous nature is complicated by its clonal structure, with multiple genetically distinct clones co-existing in the same patient [17,34,35].

LSC reflect this clonal architecture and are organized into genetically diverse co-existing subclones [17,36,37] that are in constant evolution throughout the disease progression [36].

The initial attempts to universally define the LSC phenotype as Lin−CD34+CD38− [6,24] were underestimating their phenotypic heterogeneity, potentially due, in part, to antibody-related technical artefacts [38].

Although LSC have been shown to be highly enriched within the Lin−CD34+CD38− compartment [7] and the abundance of such a compartment has been directly linked to patient outcome, survival, relapse, and minimal residual disease [39,40], recent studies revealed that LSC immunophenotypic patterns can change between clones within the same tumor and between AML subtypes.

Indeed, functionally defined LSC have been also detected within the CD34+CD38+, CD34-CD38+, and CD34-CD38- cellular fractions of AML samples. The proportions of LSC within each fraction are highly variable between patients, and LSC of different phenotypic classes are observed in the same sample for the majority of patients [7].

The notion of LSC intra-tumor multivarious immunophenotype is further supported by a recent study revealing the co-existence of at least two LSC fractions with distinct immunophenotypic and molecular signatures in more than 80% of AML samples analyzed. It was shown that one LSC fraction resembled the lymphoid-primed multipotential progenitors (LMPP) (LMPP-like LSC, Lin-CD34+CD38−CD90−CD45RA+) and the other resided within the granulocyte-macrophage progenitor compartment (GMP) (GMP-like LSC, Lin-CD34+CD38+CD123+CD45RA+). These two LSC populations shared a hierarchical relation, with LMPP-like LSC being upstream of GMP-like LSC. Consistently, LMPP-like LSC had higher frequencies of leukemia-initiating cells and a gene expression profile more similar to immature AML subtypes as compared to GMP-like LSC [41].

This discovery further fuels the existing debate related to the LSC cell of origin being attributed to HSC or committed progenitors [6,8,37,41].

Technological advances in mass cytometry provide a superior tool over flow cytometry to investigate phenotypic variations and intracellular signaling modifications occurring in LSC [42]. Using this technique, Behbehani et al. observed that the expression of several surface markers (e.g., CD7, CD33, CD34, CD38, CD45, CD47, CD71, CD99, CD117, CD123, CD321, HLA-DR) on Lin-CD34+CD38lo LSC varied according to AML genetic signature and karyotype and that different AML subtypes were associated with distinct intracellular signaling in LSC [43]. The association between phenotype and genotype in AML is further supported by other studies [17,44] reinforcing the hypothesis of a fundamental role of mutations in antigenic expression instability throughout clonal selection [17] and the prognostic potential of LSC phenotyping [44].

From a functional point of view, LSC heterogeneity is observed with regard to their self-renewal and in vivo repopulation abilities (with short-term, long-term, and quiescent long-term LSC) [8], and their quiescent state [5]. This latter can be related to the AML genetic background as a recent study showed that the proportion of proliferating LSC increased within AML samples with core-binding factor (*CBF*) mutations and sensitive to chemotherapy as opposed to poor prognosis samples carrying *FLT3-ITD* mutation and normal karyotype. These results support the hypothesis that LSC are crucial determinants of clinical outcome [43].

Importantly, LSC plasticity increases upon relapse, as a potential consequence of ineffective treatments [42].

Upon disease relapse in AML, LSC considerably increase in number (9–90-fold) and exhibit higher phenotypic variability [42] and metabolic flexibility, with higher fatty acid metabolism under amino acid shortage [31], as compared to diagnostic specimens. LSC changes upon treatment seem to be only in minor proportion linked to the acquisition of additional genetic aberrations after therapy [42]. Conversely, AML relapse seems to be associated with the selection of pre-existing drug-resistant clones rather than chemotherapy-induced mutations [36].

At least two different AML cell subsets, already present at diagnosis, have been shown to contribute to relapse: early progenitors (LSC) or more committed subsets that have acquired stemness properties. Of relevance, the cell subset giving rise to the recurrence of the disease has been linked to different AML French-American-British subtypes and could further influence the choice of treatment to adopt [36].

In conclusion, as LSC are “moving targets”, future targeted therapeutic approaches against AML are likely to shift from a universal to increasingly personalized medicine and may vary according to the stage of the disease to treat.

Future approaches designed to achieve the targeted eradication of LSC early after diagnosis may prevent their evolution into more complex targets upon treatment [5].

### 2.3. The Role of the BM Niche in AML

In physiological conditions, the BM niche is a composite organization of stromal and hematopoietic cells (including osteoblasts (OB), adipocytes, perivascular mesenchymal stem, endothelial and nervous cells, megakaryocytes, regulatory T-cells (Tregs), and phagocytes) that physically supports HSC and finely controls their self-renewal, proliferation, differentiation, and migratory activities, as extensively reviewed in [45].

In leukemia, neoplastic cells compete with healthy HSC for the BM niche occupancy, by remodeling the environment at their own advantage and creating a hostile habitat for normal hematopoietic stem and progenitor cells (HSPC) [46].

The modifications observed in the BM of AML patients at diagnosis include increased microvessel density [47], neuropathy [48], reduced frequency of CD146+CD166- mesenchymal progenitors [49], decreased OB pool size [50], and diminished adipocyte counts and size [51]. The appearance of these changes observed in the BM niche follows a specific temporal order, reflecting the AML developmental phase. The establishment of a pre-leukemic niche is followed by the development of a leukemia-permissive and lately self-reinforcing environment [52]. Some of these alterations, such as the microvessel density, the loss of nestin expression (indicator of nerve damage), and the aberrant cellular composition of the BM mesenchymal and osteoblastic compartments, have been correlated to AML clinical features, such as aggressiveness, sensitivity to treatment, and patients’ overall survival [47,48,49].

Transgenic mouse models allowing the induction of genetic mutations in distinct BM stromal cell types have revealed that alterations of the BM microenvironment are not only a result but also a potential driver of malignant transformation in HSPC, as reviewed in [53]. The role of the BM microenvironment in the pathogenesis of blood cancers is still subject to debate, although it is considered highly unlikely that it constitutes the first hit of malignant transformation [53].

Conversely, leukemia-induced functional alterations have been reported in several BM niche cellular components (including OB, nerves, adipocytes, and mesenchymal stromal cells (MSC)) in AML mouse models, recapitulating human AML-BM features. Specifically, AML cells can cause OB reduction and malfunctioning [50,54] and sympathetic neuropathy [55] in vivo, all prerequisites for leukemic spread [50,55]. Moreover, leukemic cells carry the potential to hinder the adipogenic differentiation of MSC, resulting in loss of adipocytes in the hematopoietically active red BM [51]. In a recent study using single-cell RNA sequencing (scRNA-seq), Baryawno et al. were able to finely dissect the cellular composition of the BM niche in an AML mouse model and capture the cellular and molecular trajectories taking place in the BM in the initial phase of AML development. In particular, they observed alterations in the osteogenic and adipogenic potential of MSC, arrest in OB maturation, blood vessel remodeling, increased expression of hypoxia-related genes, and decreased expression of HSC-supportive signaling factors [56], in line with the hypothesis of the remodeling of an increasingly leukemia permissive and supportive BM niche.

AML cell intrinsic properties can also profoundly alter the tumor immune microenvironment, hampering the existing anti-tumor response. AML can contribute to defective T- and natural killer (NK)-cells, by reducing the numbers of T, T helper (Th) 1, and cytotoxic T lymphocytes in favor of Tregs and Th17, and by promoting M2-like monocyte polarization [16,57,58]. The aberrant immune landscape observed in AML patients is induced by the secretion of immunosuppressive cytokines (e.g., interleukin (IL)-4 and -10 and transforming growth factor (TGF)-β) and the block of pro-inflammatory ones. T-cell inhibitory enzymes such as indoleamine 2,3-dioxygenase 1 (IDO1) and arginase, of immune checkpoints (e.g., cytotoxic T-lymphocyte antigen-4 (CTLA-4) and programmed death ligand-1 (PD-L1)) and the production of nitric oxide, galectins, and ROS have also been shown to promote an immunosuppressive microenvironment in AML [52,57,58]. Recently, using an integrated single-cell analysis Van Galen et al. showed that CD14+ monocyte-like AML cells are responsible for mediating immunomodulatory activities [16]. Additionally, the increment of myeloid-derived suppressor cells (MDSC) induced by AML cells enhances the immunosuppression in AML niche [53]. Furthermore, AML cells can preferentially evade immune surveillance by downregulating the expression of major histocompatibility complexes (MHC) and natural killer group 2 member D (NKG2D) ligands, which are required for immune recognition by T- and NK-cells, respectively [59,60,61].

Majeti et al. observed that LSC and HSC exhibit a divergent expression of pathways related to their crosstalk with the BM niche [28]. Specifically, LSC are unresponsive to Notch and TGF-β niche signaling, reported to hinder HSC growth and myeloid differentiation [62], and downregulate CXCR4-STAT3/5B signaling pathways, N-cadherin, and alpha E-catenin [28]. On the contrary, AML cells upregulate CXCR4, VLA-4, CD44, E-selectin, and CD98 adhesion molecules [45,52], and LSC rely on CXCL12/CXCR4 and integrin/OPN signaling for their adhesion and persistence, on Wnt/β-catenin and PI3K/Akt signalings for their self-renewal and maintenance [63], and on SIRPα/CD47 binding for their survival and functionality [64].

The expression of some adhesion factors (e.g., CXCR4, VLA-4, CD44v6 the most common AML CD44 isoform, G protein-coupled receptor 56 (GPR56), and junctional adhesion molecule (JAM)-C) in AML cells/LSC was associated with poor patient survival and was found increased in specific risk groups (e.g., VLA-4 in non-high-risk pediatric and adult patients, and GPR56 at mRNA level in intermediate- and high-risk patients) [65].

Cell-to-cell communication between AML and their BM microenvironment not only occurs through direct contact and exchange of soluble factors but also through exosomes [66].

The AML-BM microenvironment can also support leukemia development by providing energetic supplies and acting as a chemoprotective milieu. For instance, in response to AML stimuli, endothelial cells and adipocytes support leukemic growth and persistence by secreting granulocyte-macrophage colony-stimulating factor (GM-CSF), granulocyte colony-stimulating factor (G-CSF), IL-6, and serving as a source of fatty acids [47,67]. OB can augment neoplastic cell proliferation, through an AML-OB crosstalk involving IL-1β and GM-CSF or by inducing leukemic cells to secrete higher levels of IL-8, a pro-angiogenic factor [68].

BM-mediated chemoprotection can be promoted by several cell types, including cancer-associated fibroblasts (CAF), -through the release of growth differentiation factor 15 [69], activator of TGF-β signaling in chemo-resistant leukemic cells [70]-, and endothelial cells, through the production of vascular endothelial growth factor (VEGF) and other adhesion molecules which trigger survival and proliferative pathways in AML cells [47]. MSC can contribute to AML survival, for example, by inducing the activation of Notch [71] and c-Myc [72] signaling and increasing the expression of anti-apoptotic factors Bcl-2 and Bcl-X_L_ [73]. MSC have also been described to act as a source of functional mitochondria for leukemic cells and LSC, thus fueling their energetic consumption [74].

The hypoxic nature of AML-BM microenvironment can also contribute to AML chemoresistance by favoring quiescence of leukemic cells [75]. This is due to hypoxia-induced cell cycle arrest and pro-survival signaling, as indicated by the upregulation of p27 (a regulator of cell cycle which prevents S-phase entrance), the increased expression of XIAP (an anti-apoptotic molecule), and the pro-survival PI3K/AKT pathway activation observed in AML cells upon in vitro culture in hypoxic conditions [76].

A more extensive summary of BM-chemoprotection mechanisms is reviewed in [11].

BM immunohistopathology of leukemia xenograft models localized residual leukemic cells, after chemotherapeutic treatment, in proximity to the vascular endothelium and to the endosteum, supporting the role of the BM niche in preserving LSC from the effect of chemotherapeutic agents [10].

Overall, these peculiar features of the AML niche are likely to impact on drug efficacy and their function should be taken into account when testing novel therapies.

2/3D in vitro co-culture systems and 3D in vivo ectopic BM ossicles, which better recapitulate the complexity of the human pathologic BM, are becoming critical tools to test drug efficacy in a human-resembling environment, as reported in [77], and to identify potential new therapeutic targets involved in leukemia-stromal interactions. An exemplification of the broad applicability of in vitro BM niche models in the leukemic context was recently provided by a sophisticated leukemia-on-a-chip technology [78]. This was applied to the investigation of the B-cell acute lymphoblastic leukemia (B-ALL) microenvironment but carries great potential in AML.

Moreover, single-cell technologies are emerging as a powerful tool to dissect normal and, more importantly, pathological niches. They are allowing to understand how leukemia reshapes the BM microenvironment for the identification of cellular and molecular alterations potentially predictive of patient survival and/or targetable with specific stromal/immune microenvironment-directed therapeutic approaches [19,56].

Novel strategies investigating the preclinical/clinical utility of exploiting and targeting peculiar features of the AML niche (as hypoxia, adhesion molecules, cellular, and immune aberrations) to treat the disease are currently under investigation [22,52,53,65]. BM therapeutic targeting could not only hinder AML progression but also sustain and preserve normal hematopoiesis [51].

## 3. CAR T-Cells and Their Current Clinical Application in AML Therapy

Chimeric antigen receptor (CAR) T-cell therapy is a form of “adoptive T-cell transfer”, originally described in the late 1980s by Eshhar et al. [79,80,81]. The CAR is an artificial receptor, integrating an extracellular antigen-binding domain comprised of the variable region (variable heavy [VH] domain-linker-variable light [VL] domain) and the hinge region of an antibody fused to a transmembrane domain. The transmembrane domain is connected to cytoplasmic signal transducing chains, such as the TCR-ζ (CD3ζ) and the co-stimulatory domains (e.g., CD28, 4-1BB, ICOS, OX40), which promote T-cell activation [82]. When the CAR is expressed on the T-cell surface (CAR T-cell), it mediates a non-MHC-restricted antigen recognition coupled to T-cell activation, a property scientists exploit to target tumor antigens and eradicate cancer. In a clinical setting, patients could receive CAR T-cells derived from their own T-lymphocytes engineered ex vivo (autologous CAR T therapy) [83] or from a donor’s T-cells (allogeneic CAR T therapy) [84].

Autologous CAR T-cell therapy demonstrated promising results in a series of clinical trials against chronic lymphocytic leukemia [85,86,87], B-ALL [87,88,89,90,91,92], and diffuse large B-cell lymphoma [92,93,94,95,96,97,98,99], leading to the Food and Drug Administration (FDA) and European Medicines Agency (EMA) approval of two autologous anti-CD19 CAR T drugs, namely Kymriah™ [100,101,102] and Yescarta™ [103,104].

In the AML context, CAR T-cell application is at its dawn, with a limited number of reported clinical trials and benefits in the treated patients. The first clinical application of CAR T-cells had been reported in 2013 by Ritchie et al. targeting Lewis-Y antigen with unsatisfactory results [105]. Since then, reported AML CAR T clinical trials targeted mainly one single antigen (mostly CD33, CD123, and NKG2D ligands) [106]. Up to 2021, across all AML reported clinical trials, an estimate of 65 AML patients have been treated with CAR T products, only a quarter of whom have achieved complete remission [106]. As summarized in [106], most of the anti-CD33 CAR T-cell therapies resulted in partial responses, and no responses were observed in all 31 patients treated with anti-NKG2D ligands CAR T-cells across three studies. The most encouraging results have been reported by 3 patients who reached complete remission within one month after being infused with anti-CLL1 CAR T-cells [107].

To date, there are more than 20 ongoing AML CAR T clinical trials registered with the clinicaltrials.gov. This number is expected to grow as more target antigens are evaluated preclinically. Relevant target antigens include: CD135 [108,109,110], CD38 [111], folate receptor (FR)-β [112,113], WT1 [114,115], B7-H3 [116,117], CD70 [118,119], and CD7 [120]. For an overview on the indexed CAR T clinical trials in AML and the CAR constructs they employ, we refer the reader to the recent reviews from Mardiana and Gill [121] and Fiorenza and Turtle [106].

In the next paragraph, we discuss the main obstacles impeding CAR T-cell successful application in AML and potential strategies to overcome them.

## 4. CAR T-Cell Approaches in AML: Challenges and Novel Strategies

Although several preclinical and clinical studies are investigating CAR T-cell approaches in AML, their successful application remains challenging [122].

Beside the general limitations of CAR T-cell therapies (e.g., toxicities (cytokine release syndrome and neurotoxicity), relapse, durability of the response, and accessibility to the treatment) [123,124], CAR T exploitation in AML is further challenged by the complex pathobiology of this disease.

The obstacles limiting the application of CAR T-cells to AML include the lack of a universal and tumor-specific antigen, and an immunosuppressive tumor microenvironment, as reported for solid tumors [121,124,125].

**Figure 2:**
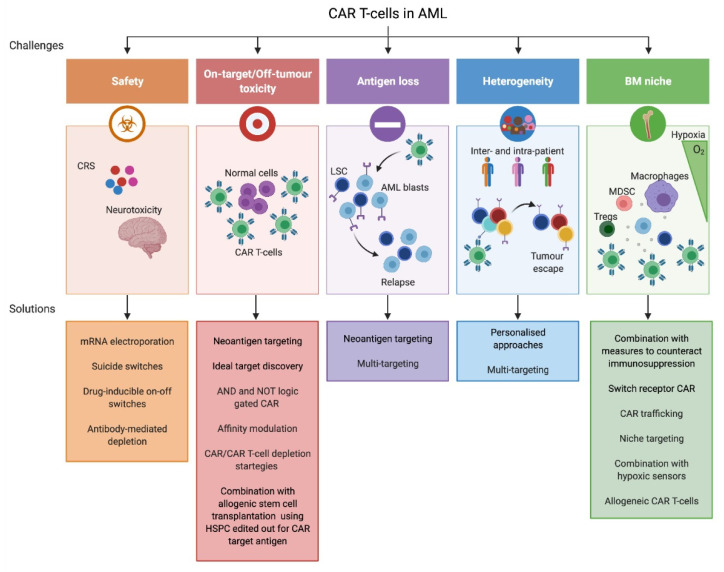
Schematization of chimeric antigen receptor (CAR) T-cell limitations in acute myeloid leukemia (AML) therapy and possible strategies to overcome them. Cytokine release syndrome (CRS) and neurotoxicity are the most frightening side effects related to the use of CAR T-cells in patients. Strategies to deplete CAR/CAR T-cells (e.g., mRNA electroporation of the CAR, suicide switches, drug-inducible on–off switches, and antibody-mediated depletion) could be employed to reduce the persistence of the toxicity. Due to the absence of an ideal target in AML, CAR T-cells impact normal cells expressing the target antigen. Thus, it is essential to identify suitable neoantigens and novel potential targets (leukemia-specific). To overcome on-target/off-tumor toxicity, AND- and NOT-logic-gated CAR can be employed to increase specificity. Affinity fine-tuning, CAR/CAR T-cell depletion strategies, and a combination of CAR therapies with allogeneic HSPC transplantation edited out for CAR target are further solutions to be considered. Antigen loss can occur upon CAR treatment. However, the targeting of neoantigens, stably expressed on leukemic cells, or of multiple antigens should overcome it. AML inter- and intra-patient heterogeneity renders extremely difficult a general application of CAR T in all AML patients, suggesting that a personalized approach and combinatorial CAR strategies are required. Lastly, AML-BM niche might affect CAR T-cell functionality due to its hypoxic and immunosuppressive nature. Moreover, it could protect LSC from CAR T-cell effectiveness. Strategies to combat immunosuppression should be considered alongside CAR infusion. Armored or switch receptor CAR can be employed as well. Specific antigen expression or hypoxia can be exploited for CAR trafficking or niche targeting increasing CAR T potency in the cradle of LSC. To overcome potential alterations of AML T-cells that can render them not suitable for CAR T-cell manufacturing, allogeneic T-cells engineered to bypass graft-versus-host disease and rejection and to express CAR can be used. CRS, cytokine release syndrome; CAR, chimeric antigen receptor; LSC, leukemic stem cells; AML, acute myeloid leukemia; HSPC; hematopoietic stem and progenitor cells; BM, bone marrow; MDSC, myeloid-derived suppressor cells; Tregs, regulatory T-cells.

In the next sections, we discuss the challenges for CAR T-cell use in AML in light of the disease characteristics: (1) identification of specific LSC/leukemic CAR target, (2) AML heterogeneity, and (3) leukemic BM niche. Limitations and potential rescue strategies are schematized in Figure 2.

### 4.1. CAR T-Cell Antigens in AML and Strategies to Overcome on-Target/Off-Tumour Effects

The primary barrier to the successful application of CAR in AML is the lack of surface targets that are specifically expressed on AML leukemic cells in the majority of patients, but not on normal tissues [126].

The vast majority of markers investigated so far are not restricted to LSC/leukemic cells but are also found on normal hematopoietic progenitors, on mature cells (e.g., CD7 on T-cells) and on non-hematopoietic sites (e.g., CD33 on Kupffer cells, CD123 on endothelial cells, CD44v6 and CD47 on keratinocytes, FLT3 on neurons and testis, CLL1 on lung and gastrointestinal epithelial cells) [122].

As a consequence, although the CAR T-cells that are being tested for AML exhibit a potent anti-leukemic effect in preclinical studies, the majority of them only show modest effectiveness in clinical trials and are associated with myeloablation, cytopenia, and severe extramedullary effects [106,121,122]. The on-target/off-tumor toxicity effects observed in AML are less tolerable than the B-cell aplasia induced by anti-CD19 CAR T-cell treatment in ALL and they can be lethal upon prolonged exposure [121,122]. Thus, clinical evidence suggests that current CAR T-cell strategies in AML could serve as a bridge to hematopoietic stem cell transplantation rather than a stand-alone therapy [127,128].

Potential strategies to avoid fatal side-effects, due to the lack of leukemia-specific antigens, include (1) discovering novel LSC/leukemic specific antigens, (2) optimizing the design of CAR T-cell treatments, (3) optimizing the design of CAR products, and (4) controlling CAR T-cell activity in vivo.

(1) To broaden the spectrum of targetable antigens, it is possible to redirect CAR T-cells to target intracellular neoepitopes when presented by HLA complex on AML cells, as exemplified by the recently developed CAR against NPM1c epitope-HLA-A2 complex [129]. Additionally, FLT3-ITD is a candidate tumor-specific CAR antigen, as it is endogenously processed by leukemic cells generating an immunogenic mutated peptide [130].

Moreover, alternative splicing-derived AML neoantigens represent (e.g., CD44v6) or may represent (e.g., FLT3-Va and NOTCH2-Va) immunotherapeutic targets [131], with CD44v6 CAR T-cells exhibiting anti-cancer killing preclinically [132] and being currently investigated in clinic (NCT04097301).

Notably, it is essential to select neoantigens expressed on the vast majority of, if not all, AML cells and patients to obtain a large applicability.

Moreover, it is unlikely that neoantigens undergo antigen loss and therefore they serve as optimal immunotherapeutic targets [131].

Epitope-HLA complex targeting may be extended to intracellular proteins selectively expressed by AML blasts/LSC, as TARP identified through differential gene expression screening [133].

As previously mentioned, scRNA-seq can be applied to the identification of novel targets to be subsequently validated at the protein level. Furthermore, the recently developed sequentially tumor-selected antibody and antigen retrieval (STAR) system has been applied to screen and identify nanobodies preferentially binding to AML cells, potentially informing leukemia-specific CAR T-cell strategies [134].

(2) It has been recently shown that CAR T-cell infusion combined with the transplantation of HSPC, in which the CAR-targeted antigen (e.g., CD33) is edited out prior to CAR infusion, can be successfully applied to avoid off-tumor toxicity. This strategy can be extended to other antigens and, after appropriate considerations, translated into patients [135].

(3) On-target/off-tumor CAR T-cell side effects can be overcome by logic-gated CAR T-cells, which require the targeting of multiple antigens in order to exert their cytotoxic effect. Specifically, “AND-gated” dual CAR T-cells can only activate when at least two markers are expressed on the target cells. Conversely, “NOT-gated” CAR T-cells require the expression of one marker and the absence of a second one to exert anti-tumor effects [124]. However, high-throughput integrated transcriptome and surface proteome analyses in AML blasts/LSC failed to identify suitable antigen combinations for the design of dual CAR strategies, due to the high heterogeneity observed in this leukemia [126]. Another study has identified CD33-TIM3 and CLL1-TIM3 as potential combinations for the development of dual CAR approaches and will require further validation [136]. Dual CD13-TIM3 CAR T-cells evaluated in preclinical settings exhibited anti-leukemic potential with limited HSC toxicity [134].

The affinity of CAR to their cognate antigens can influence on-target/off-tumor effects. The effect of CAR affinity on CAR T-cell functionality has generated controversial results [82], probably due to the antigen-dependent variability and the CAR construct utilized. Low-affinity CAR can better discriminate cells with different antigen expression levels, leading to the preferential targeting of malignant cells (overexpressing tumor antigen) over normal cells (expressing the antigen at physiological levels) [82]. Thus, their use would be ideally placed in AML, to minimize the off-leukemia drawbacks. Low-affinity CD123 CAR exhibit similar anti-leukemic properties to wild-type and high-affinity ones but a promising safer profile in vitro, as determined by reduced cytotoxicity against lowly antigen-positive cells [137]. On the other hand, while decreasing the affinity of CAR T-cells against FR-β in AML can prevent their off-tumor toxicity in monocytes, it does not provide sufficient anti-leukemic activity [113]. Further investigations will be necessary to fine-tune CAR affinities to balance anti-tumor potency and toxicity.

(4) Finally, CAR mRNA electroporation in T-cells, suicide switches, drug-inducible on–off switches, or antibody-mediated CAR T elimination are strategies to control CAR T-cell lifespan and limit their side effects [23,124,132,138,139,140]. However, their translation in clinic needs accurate evaluation [141].

### 4.2. AML Phenotypic Heterogeneity Challenges the Identification of Target Antigens for AML CAR T-Cell Strategies

The inter- and intra-patient genetic and phenotypic heterogeneity of AML limits the design of a universal CAR T-cell strategy.

As a result, the selection of candidate surface antigens requires a more personalized approach varying according to the age of the patient [142], disease phenotype [143], and stage [42,136]. In a recent study, a stable expression at diagnosis and relapse for CD33, CD123, TIM3, and CD7 has been reported [136]. Further studies investigating the stability of cell surface markers in LSC/AML blasts throughout the progression of AML are needed, especially at relapse, when CAR T-cell therapies are more likely to be utilized.

The mutational landscape can also contribute to AML immunophenotypic patterns [43]. For instance, CD123 [144] and CD26 [44] display high expression in *FLT3-ITD*-mutated LSC, NPM1c−HLA−A2 complex in NPM1c+HLA−A2+ AML [129], and CD33-FLT3 in *KMT2A*-mutated infant patients [142].

Antigen loss or reduced expression on the tumor cell surface is one of the main causes of disease relapse in CD19 CAR T-cell therapy [123]. Similarly, in AML, CAR T-cells against antigens with heterogeneous expression are likely to result in relapse due to incomplete targeting and clonal selection, especially if single antigens are targeted [127].

Overcoming this limitation is likely to require the targeting of multiple antigens (CAR/CAR strategy) [139]. Indeed, CAR T-cells concurrently targeting antigens on LSC and AML blasts (CD123, CD33) have shown remarkable preclinical results [139]. Perna et al. and Haubner et al. used transcriptomics, surface proteomics, and flow cytometry to systematically identify targets expressed in primary AML-LSC but absent in HSPC and healthy tissues [126,136]. By doing so, they identified candidate antigen combinations for CAR/CAR strategies (e.g., ADGRE2-CD33, CCR1-CLEC12A, CD70-CD33, LILRB2-CLEC12A [126], CLL1-TIM3 [136]), which will require functional validation. Dual CAR targeting (involving CD123, CD33, CLL1) is also being tested in clinical trials (NCT03795779, NCT04156256, NCT04010877). Encouraging results have been reported for CLL1-CD33 CAR T-cell treatment (NCT03795779) [145].

Moving toward a personalized medicine approach, recent clinical studies are investigating patient-tailored approaches combining CAR T-cells against various targets according to patient’s AML phenotypes (NCT03222674, NCT03473457).

### 4.3. Targeting the AML Tumour Microenvironment with CAR T-Cells

So far, there is no direct evidence of the impact of the AML niche on CAR T-cell functions. Given its immunosuppressive and hypoxic nature, it is likely that the AML-BM niche may hamper CAR T-cell functionality. Thus, CAR T-cell strategies targeting both leukemia cells and their microenvironment may represent a double-edged approach, counteracting the pro-leukemic BM microenvironment on one side and directing CAR T-cells to the LSC-enriched BM on the other.

The microenvironment-mediated impairment of CAR T-cell functions is well documented in solid tumors and B-cell malignancies, where it can be mediated by various cell types, including MDSC, macrophages, and Tregs. A similar mechanism may be extended to AML [125,146].

Despite the immunomodulatory functions of BM-MSC and their role in tumor evolution [147], few studies have investigated their effect on CAR T-cell functions and generated conflicting results, showing negative [148], positive [149], or lack of [132,150] impact. In solid tumors, CAF block T-cell infiltration [125] and contribute to immunosuppression and immunotherapy resistance [151] while supporting cancer growth [125]. These observations have led to several preclinical studies testing CAR T-cells against the fibroblast activation protein (FAP) expressed in CAF. These studies have shown encouraging anti-tumor effects but, in some cases, have also displayed severe toxicities due to the expression of FAP on healthy tissues, as summarized in [125]. To date, only one study has evaluated the role of the BM-MSC in CAR T-cell resistance in AML context [132] and further investigation is required.

To counteract the tumor-associated immunosuppressive environment, fourth-generation “armored” CAR have been engineered to produce immune-stimulatory cytokines (e.g., IL-12) [124], to release PD-1-blocking single chain variable fragments [152], or to constitutively express CD40L in order to raise endogenous anti-cancer immune response [153]. Chimeric constructs, coupling the extracellular domain of an inhibitory T-cell receptor (e.g., PD-1) with intracellular costimulatory signal, have also been described in CAR T-cells [154].

Additional strategies to counteract the tumor immunosuppressive microenvironment in the CAR T-cell context are described elsewhere [125,146].

Trafficking receptors (e.g., CXCR4) [155] may be useful to preferentially direct CAR T-cells to the BM, potentially boosting the eradication of residual LSC. Alternatively, the combination of CAR T-cells with LSC mobilizers might favor the egress of LSC from their niche [65] and their consequent killing by CAR T-cells in a non-hostile environment for CAR T-cells.

It is still to be determined whether the hypoxic nature of the AML niche positively or negatively influences CAR T-cell functions. Hypoxic environments have been shown to promote central memory phenotype in CAR T-cells, which is beneficial for their functionality [156], but on the other hand reduce CAR T proliferation and hinder their effector memory differentiation and functionality (the latter being due to Tregs recruitment and enhancement of PD-L1 expression) [125]. Moreover, the hypoxic environment can alter the surface phenotype of AML cells in vitro [157], suggesting that the effect of hypoxia should be evaluated when selecting CAR T-cell targets to avoid antigen escape. A recent study illustrates how hypoxia-sensitive CAR T-cells, able to specifically activate in hypoxic sites (such as the AML-BM microenvironment) can be employed to avoid unwanted off-site toxicities [158]. This approach could be investigated to eradicate residual LSC persisting in the hypoxic BM niche after chemotherapy.

The application of in vitro and in vivo humanized BM niche models will be crucial to understand how the AML-BM modulates CAR T-cell functions and to identify therapeutic targets in the pathological niche.

Finally, AML intrinsic features and prior chemotherapy treatments may impact on the fitness of the underlying T-cell populations used for CAR engineering, challenging the successful manufacture of autologous CAR T-cells from AML patients [121]. Allogeneic CAR T-cells engineered to attenuate graft-versus-host disease and rejection may be employed to achieve faster and broader product availability [159]. A phase I clinical study is currently investigating the applicability of universal CD123 CAR T-cells in AML (NCT03190278).

## 5. Conclusions

The complexity of AML biology has led to unsatisfactory clinical outcomes so far. The quiescent and immune-evasive nature of LSC makes them critical players in therapy escape and disease relapse, suggesting that their effective targeting is imperative for curative treatments. In this context, CAR T-cells represent a promising option, as they can effectively target tumor cells irrespectively of their quiescent status or their immune visibility, by mediating MHC-independent tumor recognition and targeting.

In this review, we summarized the main characteristics of AML-LSC and the AML-BM niche in the context of their therapeutic targeting using CAR T-cells. Moreover, we highlighted several potential strategies to minimize toxicity while preserving or increasing CAR T-cell functions.

Although CAR T-cells have been successfully applied for the treatment of several hematological malignancies and their translation to AML is in its infancy, it is clear that the pathobiology of the disease represents the main barrier to their successful exploitation. Specifically, the lack of LSC/AML-specific target antigens, the heterogeneity of the disease and the potential role of the AML pathologic BM microenvironment stand out as some of the main obstacles. Additionally, AML patients’ age and their general compromised health status increase the risks of CAR T-cell related toxicities.

It is increasingly clear that the application of CAR T-cells to AML will require personalized and multi-targeted approaches and these strategies are currently investigated in clinical trials.

Luckily, the CAR T-cell research field is continuously evolving and there is still room for improvement. In particular, recent single-cell technologies can be applied to identify novel candidate antigen combinations in AML cells and their niche. The advances in CAR engineering and the introduction of control switches will lead to increased CAR activity and specificity and reduced toxicity.

Open questions remain: (1) Is there a broadly applicable antigen combination across AML subtypes? (2) Are some of the emerging strategies (e.g., CAR depletion, niche-targeting) feasible in a clinical setting? (3) Could CAR T-cells ever be used as a stand-alone therapy in AML? (4) Can stratification systems be developed to identify patients eligible for CAR T-cells as a first-line treatment?
